# Ca^+^ Ions Solvated in Helium Clusters

**DOI:** 10.3390/molecules26123642

**Published:** 2021-06-15

**Authors:** Massimiliano Bartolomei, Paul Martini, Ricardo Pérez de Tudela, Tomás González-Lezana, Marta I. Hernández, José Campos-Martínez, Javier Hernández-Rojas, José Bretón, Paul Scheier

**Affiliations:** 1 Instituto de Física Fundamental (IFF-CSIC), Serrano 123, 28006 Madrid, Spain; maxbart@iff.csic.es (M.B.); j.campos.martinez@csic.es (J.C.-M.); 2 Institut für Ionenphysik und Angewandte Physik, Universität Innsbruck, Technikerstr. 25, A-6020 Innsbruck, Austria; Paul.Martini@uibk.ac.at; 3 Lehrstuhl für Theoretische Chemie, Ruhr-Universität Bochum, 44780 Bochum, Germany; ricardo.perez@theochem.rub.de; 4 Departamento de Física and IUdEA, Universidad de La Laguna, 38205 Tenerife, Spain; jhrojas@ull.es (J.H.-R.); jbreton@ull.edu.es (J.B.)

**Keywords:** molecular clusters, solvation, helium-alkaline earth ion interactions, helium nanodroplets, mass spectrometry, classical/quantum monte carlo calculations

## Abstract

We present a combined experimental and theoretical investigation on Ca${}^{+}$ ions in helium droplets, He${}_{N}$Ca${}^{+}$. The clusters have been formed in the laboratory by means of electron-impact ionization of Ca-doped helium nanodroplets. Energies and structures of such complexes have been computed using various approaches such as path integral Monte Carlo, diffusion Monte Carlo and basin-hopping methods. The potential energy functions employed in these calculations consist of analytical expressions following an improved Lennard-Jones formula whose parameters are fine-tuned by exploiting ab initio estimations. Ion yields of He${}_{N}$Ca${}^{+}$ -obtained via high-resolution mass spectrometry- generally decrease with *N* with a more pronounced drop between N=17 and N=25, the computed quantum He${}_{N}$Ca${}^{+}$ evaporation energies resembling this behavior. The analysis of the energies and structures reveals that covering Ca${}^{+}$ with 17 He atoms leads to a cluster with one of the smallest energies per atom. As new atoms are added, they continue to fill the first shell at the expense of reducing its stability, until N=25, which corresponds to the maximum number of atoms in that shell. Behavior of the evaporation energies and radial densities suggests liquid-like cluster structures.

## 1. Introduction

Alkaline and alkaline-earth ions embedded in helium have been frequently used to investigate the superfluid properties of this substance as well as the microscopic structure of the He solvation shells around impurities. Pioneering work on these systems was done by Glaberson and Johnson who developed a technique for introducing these impurities into liquid helium and measuring their mobility [1]. They found quite different mobilities between alkaline and alkaline-earth ions, in contrast with the expectation from the Atkins snowball model [2], which assumes that the mobility should be equal as all ions have the same charge. Cole and Bachman [3] then refined the Atkins model by including the effect of the electronic structure of the core ion and the resulting approach predicted a liquid-like bubble structure for Ca${}^{+}$, Sr${}^{+}$ and Ba${}^{+}$, similar to an electron bubble, due to the effect of the remaining valence electron in the outermost orbital. Subsequent mobility measurements in superfluid helium for Be${}^{+}$, Mg${}^{+}$, Ca${}^{+}$, Sr${}^{+}$ Ba${}^{+}$ in the 1.27 K <T< 1.66 K temperature range [4] confirmed these liquid-like structures for all these alkaline-earth ions with the exception of Be${}^{+}$, whose mobility suggested a snowball structure. More recently, ground state path integral Monte Carlo (PIMC) calculations of Paolini et al. [5] and time-dependent bosonic density functional theory (DFT) simulations of Fiedler et al. [6] of various ions in He have found that Be${}^{+}$ generates snowball structures similar to those of the alkali ions, in contrast with more liquid-like structures for the heavier alkaline-earth ions, in agreement with the experiments. In these simulations, the employed ion-He interaction potentials were based on ab initio calculations. In the particular case of the He-Ca${}^{+}$ interaction, besides the coupled clusters calculations performed by Fiedler et al. [6], it is worth mentioning a DFT analysis of complexes formed with Ca${}^{+}$ and up to four atoms of He [7] and a pseudopotential treatment to obtain the corresponding potential energy curves [8].

In recent decades, helium nanodroplets (HNDs) have proven to be a well-suited platform to investigate features observed in bulk helium, such as superfluidity [9]. These finite systems offer some advantages in comparison to bulk helium such as the absence of both physical walls and photodegradation [10]. In addition, HNDs constitute a powerful spectroscopic matrix to study a variety of atomic and molecular species [11]. In particular, many studies have addressed the solvation of alkali ions using this technique [10,12,13,14,15,16,17]. For alkali-earth ions, although various theoretical simulations of their solvation in He have been reported [5,6,18,19,20,21,22], experimental studies are very scarce [23].

In this paper, we present a combined experimental and theoretical study on He${}_{N}$Ca${}^{+}$ clusters, following a similar spirit of previous investigations on either helium or molecular hydrogen complexes doped with Li${}^{+}$ or Cs${}^{+}$ ions [16,17,24]. Measurements of ion yield abundances after ionization of doped HNDs are analyzed along with theoretical calculations performed with PIMC, diffusion Monte Carlo (DMC) and basin-hopping (BH) methods for both energies and structures of He${}_{N}$Ca${}^{+}$ clusters. A new potential energy surface (PES) is employed, developed with an improved Lennard-Jones (ILJ) fit of ab initio points.

The structure of the paper is as follows: In Section 2, we present the essential details of the experimental setup (Section 2.1), the PES employed in this work (Section 2.2) and briefly describe the procedure to calculate the energies and structures of the clusters under study (Section 2.3). Results are shown and discussed in Section 3 and finally the conclusions are listed in Section 4. Details of the calculations as well as some additional figures and one table are given in the Supplementary Materials (SM) file.

## 2. Materials and Methods

### 2.1. Experimental Details

**Figure 1:**
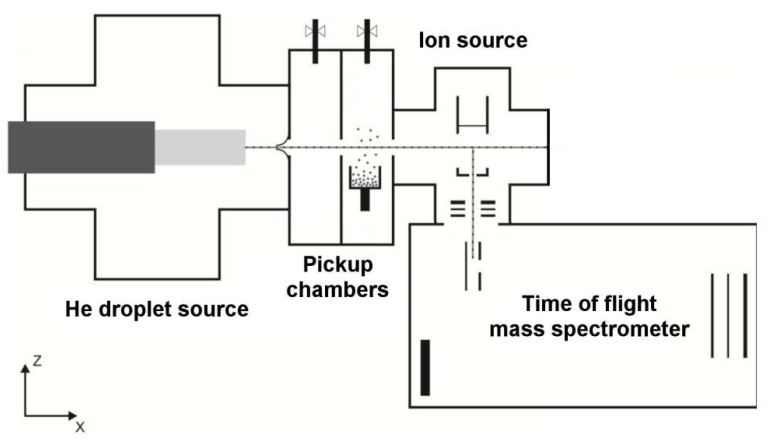
Schematic illustration of the experimental setup. Calcium vapor is picked up by neutral helium nanodroplets that are formed by expansion of precooled and pressurized helium into vacuum. The doped helium nanodroplets are ionized via electron impact and all product ions ejected from the heavy droplets are analyzed by a reflectron time-of-flight mass spectrometer.

HNDs were produced by expansion of ultrapure helium (99.9999%, Messer, Bad Soden, Germany) at a stagnation pressure of 2 MPa and a temperature of 9.65 K through a 5 μm pinhole nozzle into vacuum. The temperature of the droplet source was achieved by a closed cycle cryo cooler (Sumitomo Heavy Industries, Model RDK-415D, Tokyo, Japan) and counterheating with an ohmic resistor in combination with a PID temperature controller (Lakeshore Model 331). Without droplet production the vacuum in this section of the instrument is below 10${}^{-6}$ Pa and rises with the helium to 283 mPa. According to Gomez et al. [25] the resulting average droplet size is about 2.5 $\times \;10^{5}$ He atoms for the present conditions. The expanding He jet is passing a molecular beam skimmer (Beam Dynamics, Inc., Dallas, TX, USA) with a diameter of 0.8 mm, located 5 mm downstream the nozzle. This prevents destruction of the droplets via collisions with shock fronts and reduces the pressure in the following differentially pumped section. The pressure behind the skimmer drops to 47 mPa and is further reduced to 1.2 mPa in the next differentially pumped chamber where Ca vapor is picked up by the HNDs passing an ohmically heated oven containing metallic Ca (99.9999, Alfa Aesar, Haverhill, MA, USA), operated at a temperature of 857 K. The vapor pressure of calcium was set to a value to achieve pickup of up to a few calcium atoms. After the pick-up region, the neutral doped helium nanodroplets pass a 2.5 mm aperture into another differentially pumped chamber (pressure 3.8 $\times \;10^{-6}$ Pa) where they are ionized by electron impact. Electrons are emitted from a tungsten rhenium (W75, Re25) filament in a Nier-type ion source and extracted orthogonally with respect to the direction of the HNDs. The electron energy can be set from 0 eV to 150 eV with a measured electron trap current between 0 μA and 500 μA. In the case of the present calcium measurement optimum signal was achieved using an electron energy of 60 eV and an electron current of 225 μA. Low-mass ions ejected from the large droplets are extracted orthogonally to the flight path of the HNDs and the electron beam by weak electrostatic fields and guided into the extraction region of a high resolution reflectron time of flight mass spectrometer (H-Tof, Tofwerke) via a stack of einzel lenses. In order to separate ${}^{40}$Ca${}^{+}$ from ${}^{4}$He${}_{10}^{+}$ we operated the mass spectrometer with a resolving power of 3600. Figure 1 shows schematically the experimental setup which was described in detail previously [26].

Calcium and all heavier alkaline earth atoms are heliophobic species that reside on dimples at the surface of helium droplets [27,28]. A combined theoretical and experimental study on a single calcium atom in a mixed ${}^{3}$He/${}^{4}$He cluster reveals the distinct feature that for specific ${}^{3}$He concentrations, the Ca atoms sit at the ${}^{3}$He/${}^{4}$He interface [29]. A simple model by Stark and Kresin predicts a critical size for alkali clusters for submersion into liquid helium [30]. An der Lan et al. confirmed these values for sodium [31] and potassium [32] experimentally. Calcium is less heliophobic than the alkali metals and thus should submerge into helium droplets at smaller cluster sizes than sodium which requires a cluster size of 20 atoms. The probability for hitting the dopant cluster by the electron beam is negligibly small compared to the ionization of a He atom.

Resonant hole hopping [33,34] and ion induced dipole interaction drives the charge towards the dopant cluster. Charge transfer from He${}^{+}$ or He${}_{2}^{+}$, the latter will be formed if the dopant is not close enough to be reached by about ten hops, to the dopant cluster can lead to the formation of singly and doubly charged calcium cluster ions. The first ionization energy of Ca is 6.11 eV and the second ionization energy 11.87 eV [35]. Thus, 17.98 eV are required to form an atomic calcium dication which suggests efficient formation of Ca${}^{2+}$ ions upon charge transfer from He${}^{+}$ or He${}_{2}^{+}$. However, in a cluster, electron transfer from a neighboring calcium atom to the dication is energetically favorable and the charges will localize at opposite sites of the dopant cluster. Furthermore, calcium clusters smaller than the critical size of $n_{c}=8$ [36] will split by Coulomb explosion into singly charged fragments. Since no cluster ions He${}_{N}$Ca${}_{n}^{2+}$ with n<8 are observed, we conclude that the critical size is not modified by solvation with helium. In a recent study Laimer et al. demonstrated that large HNDs can become highly charged without fast fission into lower charged fragments [37]. The critical size for a doubly charged HND was determined to be only 10${}^{5}$ and a droplet containing 2.5 $\times \;10^{5}$ He atoms can accommodate up to four charges. All ions are solvated in liquid helium and only if the local charge density is high enough that Coulomb repulsion exceeds the binding energy of an ion to the charged HND, a low-mass ion will be ejected. The solvation energy of a dication is higher than for a monocation and therefore, preferentially singly charged ions will be ejected. On their way out of the HND, ions may still be solvated with a few He atoms. In the present study, we focus on singly charged calcium cations that are complexed with a few He atoms and from intensity drops we determine the size of the first solvation layer.

**Figure 2:**
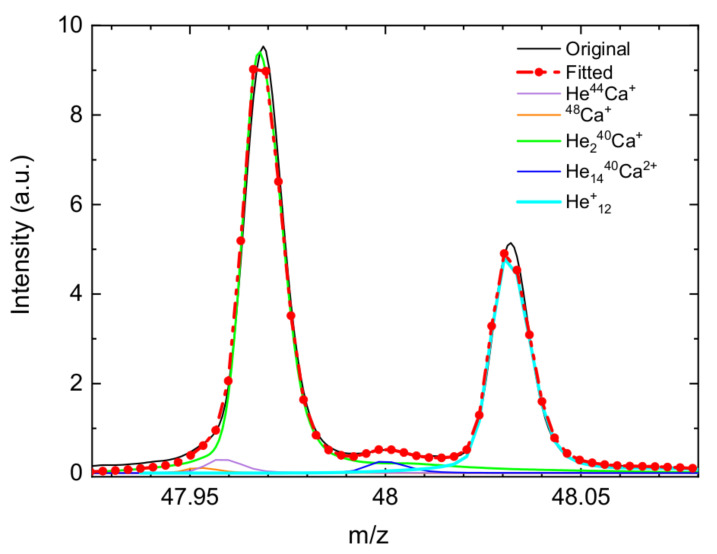
Well resolved mass spectra (arb. units) around the mass-to-charge ratio m/z=48 showing the contribution of cations that have to be considered for fitting the measured signal.

Figure 2 shows a section of the mass spectrum close to the mass-to-charge region m/z = 48 (m/z is the dimensionless quantity formed by dividing the mass number of an ion by its charge number [38]). The Figure includes a fit obtained using the custom-designed software IsotopeFit [39], from which the yields of the species of interest are extracted. The analysis of the contribution from different ions reveals the presence of bare ions such as ${}^{48}$Ca${}^{+}$ or He${}_{12}^{+}$ and combinations which also involve the dication Ca${}^{2+}$. In particular, in the example of Figure 2 the two peaks have their origin mainly in the contribution coming from He${}_{2}^{40}$Ca${}^{+}$ (maximum at the left) and from He${}_{12}^{+}$ (maximum at the right).

### 2.2. Potential Energy Surface

Pairwise two-body (2B) functions have been used to represent the He-He and He-Ca${}^{+}$ interactions. In particular, for He-He, we have employed the potential reported in Reference [40], whereas for the He-Ca${}^{+}$ contribution, a new analytical PES has been developed based on accurate coupled cluster with single and double and perturbative triple excitations [CCSD(T)] calculations and using the d-aug-cc-pV6Z [41] and def2-AQZVPP [42] basis sets for He and Ca${}^{+}$, respectively. We have checked that the adopted basis set is sufficiently large to guarantee well converged interaction energies, which are found to deviate by less than 1% from those carried out in the global minimum region with the d-aug-cc-pV5Z/def2-AQZVPP set. The CCSD(T) computations have been performed using the Molpro2012.1 package [43] and all interaction energies have been corrected for the basis superposition error by applying the counterpoise method of Boys and Bernardi [44].

From these calculations, an interaction energy of −4.453 meV is obtained at 4.285 Å, the estimated equilibrium distance. Fiedler et al. [6] reported −3.8 meV (at 4.4 Å) using the same level of theory and a less extended basis set. We have checked that the origin of the difference mostly stems from the use of a quite less extended basis set (def2-QZVPPD) for the He atom. Other works, such as the DFT study of Jalbout et al. [7] and the pseudopotential calculations of Czuchaj et al. [8], have reported He-Ca${}^{+}$ interaction energies of −5.5 and −3.7 meV at the equilibrium distances of 4.0 and 4.4 Å, respectively.

For the analytical representation of the force field, the ILJ formulation [45] of the atom-atom interactions has been chosen:

(1) $$V\left(R\right)=\epsilon \left[\frac{m}{n(R)\;-\;m}{\left(\frac{R_{m}}{R}\right)}^{n(R)}\;-\frac{n(R)}{n(R)-m}{\left(\frac{R_{m}}{R}\right)}^{m}\right].$$

**Figure 3:**
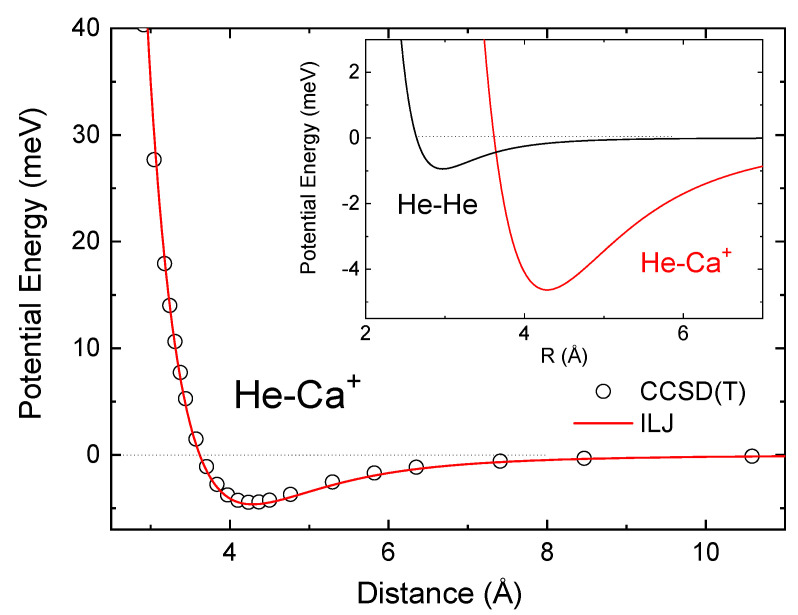
Potential energy curves for the He-Ca${}^{+}$ interaction. Open black circles are CCSD(T) results and the red line depicts the analytical expression given by the ILJ potential of Equation (1). Inset: Comparison of the well regions of He-Ca${}^{+}$ (red) and He-He [40] (black) interaction potentials.

This formulation guarantees the correct behaviour of the interaction potential in the first repulsive part, well and asymptotic regions (see Figure 3) which are those basically needed for the determination of the minima configurations of the clusters of interest. Alternative representations of the interaction, such as the Tang-Toennies potential form [46], could have been also used to this aim. However, we have preferred the former representation since it exploits a simpler analytical formula, depending on fewer and physically meaningful parameters. In the expression above, ε is the potential depth, $R_{m}$ is the minimum potential position, and n(R) is defined as follows [45]:

(2) $$n\left(R\right)=\beta +4{\left(\frac{R}{R_{m}}\right)}^{2}.$$

Values for the parameters used in Equations (1) and (2) are $R_{m}=$ 4.2851 Å, ε= 4.6308 meV, β= 5.5, and m=4. Notice that ε slightly differs from the ab initio estimation reported above; however, these are the best values for an optimum global agreement with the CCSD(T) calculations. Indeed, as can be seen in Figure 3, the analytical representation is in good agreement with the ab initio points at all intermolecular separations. It is worth noting the weakness of the interaction and how rapidly it becomes repulsive for distances shorter than ∼3.5 Å.

A comparison between He-Ca${}^{+}$ and He-He potentials is provided in the inset of Figure 3. Although the He-He interaction is weaker, as expected, both are of the same order of magnitude. Moreover, comparison with the well depths of other similar systems we have studied so far (He-Li${}^{+}$: 81.3 meV [16]; He-Cs${}^{+}$: 13.7 meV [17]) reveals that He-Ca${}^{+}$ is the weakest He-ion interaction treated in this series of combined experimental and theoretical investigations. It is well known that He interacts much more weakly with alkaline earth than with alkaline ions. Indeed, the involved dissociation energies with Be${}^{+}$, Mg${}^{+}$, Sr${}^{+}$, Ba${}^{+}$ are about 14, 8, 3 and 2 meV, respectively [6,47].

In this work, we neglect three-body effects involving the interaction between dipoles in He atoms induced by the cation [16], since typical He-Ca${}^{+}$ distances are quite large and this leads to negligible strengths of the He induced dipoles. Indeed, we have performed ab initio calculations of the He${}_{2}$Ca${}^{+}$ cluster and have found that three-body effects account for less than 1% around the minimum geometry of that cluster and that this percentage slightly increases (∼2%) for more compact configurations of the trimer.

### 2.3. Calculations of Cluster Energies and Structures

Using the previous He-He and He-Ca${}^{+}$ potentials, energies, $E_{N}$, and structures of the He${}_{N}$Ca${}^{+}$ clusters have been obtained by a combination of methods. Firstly, the unbiased BH global optimization technique [48,49] was applied to find the putative global minima of the PES for each cluster size *N*. Secondly, the cluster geometries at these minima were then used as initial arrangements for running two types of quantum Monte Carlo calculations, namely, DMC and PIMC. In the DMC approach, the ground state energies are obtained by means of a random-walk method of propagation of the Schrödinger equation in imaginary time [50,51], using the code developed by Sandler and Buch [52,53]. Moreover, the PIMC method allows us to compute the properties of the system at a given temperature [16,54]. In the present application, T= 1 K, so that results from both DMC and PIMC calculations are expected to bring similar results. Details on these methods and on their specific application to this system are given in the SM.

Cluster energies resulting from both PIMC and DMC calculations are reported in Table S1 of the SM. They compare very well for the smaller clusters, N<17, DMC energies being slightly below the PIMC ones, as corresponds to a ground-state calculation compared to a computation at T=1 K. However, for N≥ 17, DMC energies, despite following a similar behavior to that of PIMC, become higher than the PIMC ones. We have seen that this is due to an artificial evaporation of He atoms which leads to an overestimation of the DMC energies. A frequently employed procedure to avoid this issue is the use of a trial wave function [55,56,57], which, in general, is expected to yield more accurate results. Due to these difficulties, results of the DMC calculations are only reported in the following Section for the smaller cluster sizes.

## 3. Results and Discussion

**Figure 4:**
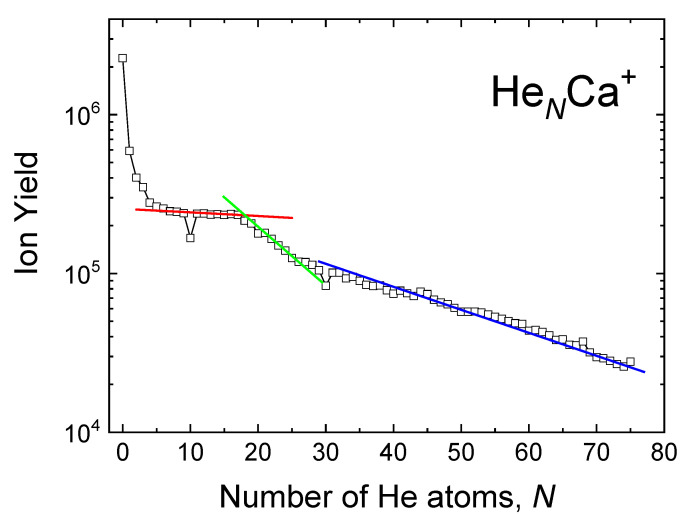
Experimental ion yield for He${}_{N}$Ca${}^{+}$. Lines red, green and blue are a guide to the eye of three regions of cluster sizes with distinct behavior.

The ion yield obtained as integrated counts for the different He${}_{N}$Ca${}^{+}$ clusters is shown in Figure 4 up to N=75. After a pronounced drop off at the smallest values of *N*, the curve exhibits a noticeable plateau between N=7 and 17 (red line therein), followed by a monotonically decreasing behavior up to N=25 (green line), whereas for N>25, monotonic decrease continues but at a perceptibly smaller rate (blue line). With the only exception of two local minima at N=10 and N=30, no other remarkable feature is observed. These anomalies originate from the huge contribution of ${}^{40}$Ca${}_{2}^{+}$ to He${}_{10}$${}^{40}$Ca${}^{+}$ and ${}^{40}$Ca${}_{4}^{+}$ to He${}_{30}$${}^{40}$Ca${}^{+}$. In general, it is very challenging to determine the right abundance of He${}_{N}$Ca${}^{+}$ clusters with *N* being a multiple of 10, due to the mentioned contributions of clusters with similar m/z ratio.

**Figure 5:**
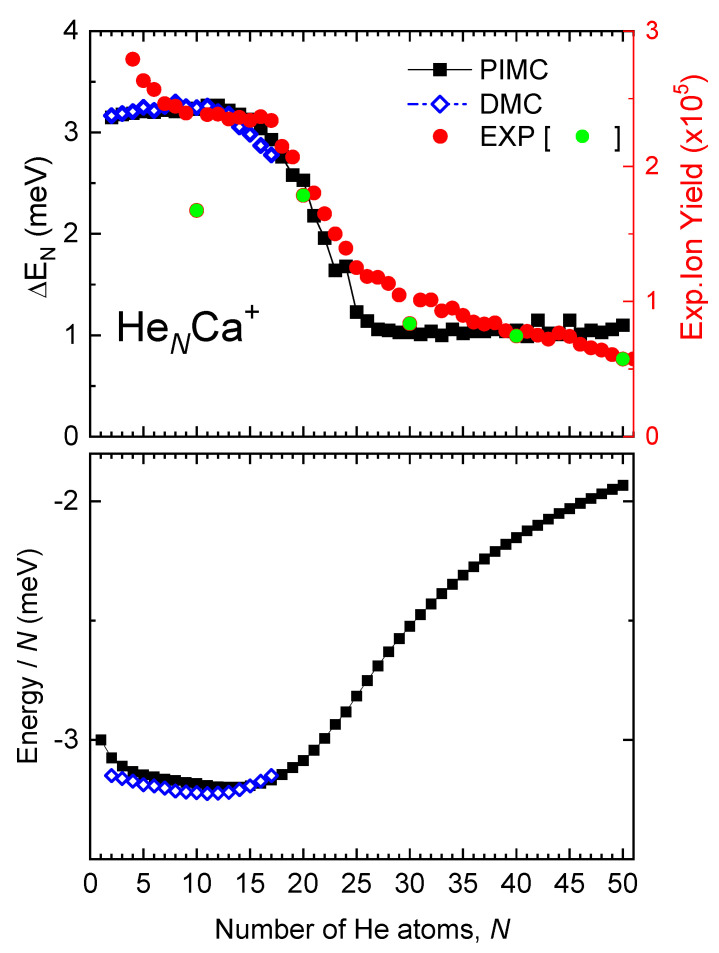
Upper panel: Evaporation energies ($\Delta E_{N}=E_{N-1}-E_{N}$) for He${}_{N}$Ca${}^{+}$ obtained with PIMC (black squares) and DMC (blue open diamonds) calculations are compared with experimental ion yields (red circles, except for *N* multiples of 10 which are shown in green, see text). Left and right *y* axes show the scales of the theoretical and experimental results, respectively. Lower panel: Energy per He atom ($E_{N}/N$) obtained by the PIMC (black squares) and DMC (open blue diamonds) approaches.

A connection between experiment and theory can be established resorting to the model of the evaporative ensemble [58]. Although there are various factors affecting the overall shape of the observed ion yield distribution, such as the size distribution of the neutral precursors and details of the ionization process, stabilities of the clusters at particular sizes can also be the reason for some features of the measured distribution [10]. Cluster stabilities are determined by means of the evaporation energies, $\Delta E_{N}=E_{N-1}-E_{N}$ (the energy required to adiabatically remove the most weakly bound atom in a cluster of size *N*). Present experiments meet the conditions of the model since, after the violent ionization process in the HNDs, the ions undergo a rapid process of fragmentation and cooling, so that the mass spectra is the result of multiple events of evaporation of the clusters. Moreover, it has been recently found that a proportional relationship between ion abundances and evaporation energies works well for weakly bound clusters as He${}_{N}$Na(K)${}^{+}$ [15], (D${}_{2}$)${}_{N}$Cs${}^{+}$ [24] and He${}_{N}$Ar${}^{+}$ [10]. In the upper panel of Figure 5 we report PIMC evaporation energies compared with the ion abundances for cluster sizes N≤51. It should be noted that the measured abundances for clusters with *N* multiple of 10 are shown in Figure 5 with a different color due to the mentioned difficulties in assigning their right values and that, on the other hand, the corresponding evaporation energies are smooth around these sizes. It can be seen that the PIMC results agree well with experiment regarding the step-like feature between N=17 and N=25: both quantities are almost constant for N= 5–17 and then, they decrease more rapidly until N≈25. For N<5 and N>25, the experimental ion yield shows more rapid variations with *N* than the PIMC evaporation energies, probably due to other factors, mentioned above, that govern its global envelope. Finally, DMC evaporation energies (also shown in Figure 5) are quite close to PIMC ones in the reported range of cluster sizes (up to N=17).

**Figure 6:**
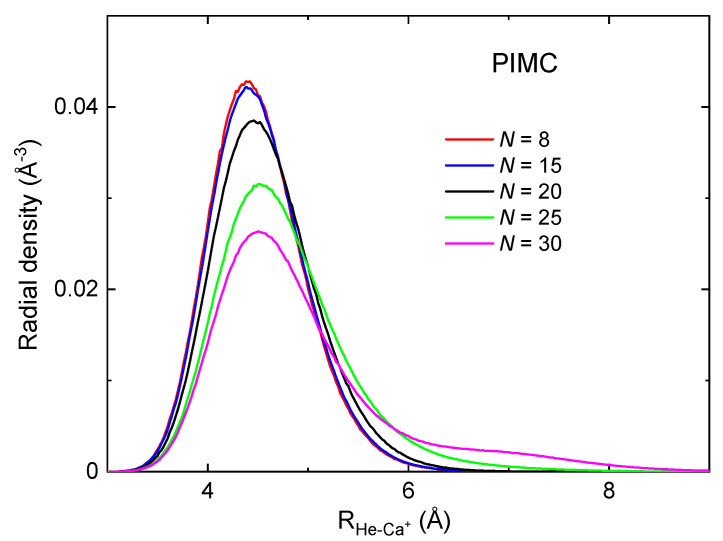
Cluster radial densities (in Å${}^{-3}$) as functions of the He${}_{N}$Ca${}^{+}$ distance (in Å) obtained by means of the PIMC method for different sizes of He${}_{N}$Ca${}^{+}$ clusters: N=8 (red), N=15 (blue), N=20 (black), N=25 (green) and N=30 (pink).

The almost constant PIMC evaporation energy (≈1 meV) for *N*= 26–50 suggests that a second solvation shell is formed for N>25, wherein the He atoms become more weakly bound. Analysis of the cluster structures provides insight into this issue. In the upper panel of Figure 6, PIMC radial densities vs. the He-Ca${}^{+}$ pair distance, $R_{{\mathrm{He}-\mathrm{Ca}}^{+}}$, are depicted for selected cluster sizes (*N* = 8, 15, 20, 25 and 30). It can be seen that for *N* = 8, 15 and 20 there is just one shell of He atoms surrounding the cation, with a wide distribution about the peak at $R_{{\mathrm{He}-\mathrm{Ca}}^{+}}\approx$ 4.5 Å. For N=25, the radial distribution becomes wider and shifted towards slightly larger values of $R_{{\mathrm{He}-\mathrm{Ca}}^{+}}$, with a tail suggesting the onset of a second shell. For N=30, this shell is distinctly seen for distances of $R_{{\mathrm{He}-\mathrm{Ca}}^{+}}\approx$ 7 Å. Hence, these radial density distributions appear to be consistent with an onset of a second shell for N>25. Dependence of the radial densities with the He-He distance is provided in Figure S3 of the SM, where the same quantities obtained from the DMC calculations are also given for the sake of completeness.

It is worth mentioning some similarities between the present system and He${}_{N}$Pb${}^{+}$ clusters, studied by DMC and also characterized by a relatively weak He-ion interaction [59]. He${}_{N}$Pb${}^{+}$ evaporation energies (Figure 5 of Ref. [59]) are nearly constant until about N=10, then they smoothly vary up to about N=17 and for larger sizes they become nearly constant again, indicating that the first shell is closed at N=17. The radial density of N=17 (Figure 7 of Ref. [59]) shows a small tail in the region where the second shell is being formed (as the distribution obtained here for N=25) and finally at N=25 a “bulge” in this region is formed, similar to the radial density we find for He${}_{30}$Ca${}^{+}$.

One can additionally ask why the evaporation energies of Figure 5 keep an almost constant value of about 3 meV for N< 17 and later undergo a gradual reduction to 1 meV in the range *N*=17–25. The constant value of the evaporation energy suggests that the He atoms occupy equivalent positions around the cation for the different sizes up to N=17. A decrease of the evaporation energy beyond that size indicates that the clusters are becoming less stable. This behavior can be alternatively noticed by studying the dependence with *N* of the energies per atom, $E_{N}/N$, shown in the lower panel of Figure 5 (see Figure S4 of the SM for a comparison with BH energies). There it is seen that $E_{N}/N$ smoothly decreases from N=1 to N≈17, the decrease being due to the small contribution of attractive He-He interactions. For N>17, this quantity starts increasing, although a second shell has not been formed yet. We have noticed that for *N*=17–25 the peaks of the density distributions become slightly but increasingly shifted towards larger values of $R_{{\mathrm{He}-\mathrm{Ca}}^{+}}$ (Figure 6). In this way, the shell can host more He atoms without significantly reducing the average He-He distances which would make the involved interactions too repulsive. This shift toward slightly larger $R_{{\mathrm{He}-\mathrm{Ca}}^{+}}$ distances (possibly together with a less attractive He-He interaction in average) can be at the origin of the increase of $E_{N}/N$ in the range *N*=17–25. Alternatively, the evaporation energy decreases with *N* in that range because less energy is required to detach an atom that is gradually less stable in the first shell.

At this point and despite the highly quantum character of these clusters, we can bring the concepts of optimal and maximum packings which are widely used in the literature of classical clusters. For instance, in the solvation of C${}_{60}$ by Ar [60] it has been found that, whereas Ar${}_{44}$C${}_{60}$ is the cluster with the lowest energy per atom (optimal packing), the system can hold four supplementary Ar atoms in the first solvation shell by slightly rising this $E_{N}/N$. Therefore, the maximum packing of the first shell is attained with 48 Ar atoms. In the present case, the optimum packing of He${}_{N}$Ca${}^{+}$ is found for N≈ 17 while the maximum packing of the first shell corresponds to the He${}_{25}$Ca${}^{+}$ cluster.

It is worthwhile to compare the results just reported with previous works [16,17] where analogous experimental-theoretical studies were carried out for the He${}_{N}$Li${}^{+}$ and He${}_{N}$Cs${}^{+}$ clusters. For He${}_{N}$Li${}^{+}$ [16], where the He-Li${}^{+}$ pair interaction is much more attractive (binding energy of the dimer of 81 meV), the N=6 and N=8 clusters were found to be particularly stable, in agreement with the experimental measurements, and the first shell, with eight He atoms, was found to be quite solid-like. Interestingly, the evaporation energies vs. *N* exhibit a drop along the *N* = 6–8 range, in contrast with the present system, where the evaporation energies gradually decrease along a larger range of cluster sizes. The behavior of He${}_{N}$Ca${}^{+}$ appears to be much closer to the features reported for the He${}_{N}$Cs${}^{+}$ cluster [17], with a smaller binding energy of the He-Cs${}^{+}$ dimer (14 meV) and a noticeable fluid-like behavior. In that system a quite smooth transition before the formation of the second shell was noticed, with the evaporation energies steadily decreasing from 10 meV at N=12 to 2 meV at N=19, keeping the latter value for N>19 clusters. This behavior was found in qualitative agreement with the experiments. The present system, with an even smaller He-ion energy (4.6 meV) presents quite similar features to those of He${}_{N}$Cs${}^{+}$ but in a much more pronounced manner: a smoother transition of the evaporation energies before the first shell is closed, a smaller $\Delta E_{N}$ for the atoms of the second shell (1 meV), and a more diffuse He-ion radial distribution for the larger clusters (Figure 6 and Figure S3 of the SM). This behavior is consistent with previous conclusions about a liquid-like bubble structure of the He${}_{N}$Ca${}^{+}$ clusters as suggested from mobility measurements [4] in bulk He as well as from theoretical simulations [3,5,6].

## 4. Conclusions

High resolution mass spectrometry and a series of theoretical approaches have been applied to study the stability and structure of helium clusters around Ca${}^{+}$ ions, He${}_{N}$Ca${}^{+}$. A good agreement has been found between the experimental yield distribution as a function of the size of the cluster and theoretical predictions obtained by means of PIMC and DMC methods. A steplike structure found both from the cluster abundances and the theoretical evaporation energies indicates that the closure of the first solvation shell gradually occurs in the range from N=17 to N=25, where a maximum of He atoms in the shell is attained. The analysis of the corresponding radial density distributions for both the He-Ca${}^{+}$ and He-He distances suggests a rather floppy, liquid-like behavior of the He atoms. These features are ultimately due to the weakness of He-Ca${}^{+}$ interaction.

## Data Availability

Data are available on the request of the corresponding authors.

## Supplementary Materials

The following materials are available online: details on classical and quantum Monte Carlo calculations; Figure S1: convergence analysis of the DMC calculations; Figure S2: convergence analysis of the PIMC calculations; Figure S3: cluster radial densities as functions of He-Ca${}^{+}$ and He-He distances (PIMC and DMC); Figure S4: He${}_{N}$Ca${}^{+}$ energies per He atom as functions of *N* (BH, PIMC and DMC); Table S1: list of He${}_{N}$Ca${}^{+}$ energies (PIMC and DMC).

## Abbreviations

The following abbreviations are used in this manuscript:

BH	Basin-Hopping
DMC	Diffusion Monte Carlo
HND	Helium nanodroplet
ILJ	Improved Lennard-Jones
PES	Potential energy surface
PIMC	Path integral Monte Carlo
