# Supplementary material for: Ca+ Ions Solvated in Helium Clusters
Source: Molecules. 2021 Jun 15;26(12):3642. doi: 10.3390/molecules26123642 (PMC8232145; doi:10.3390/molecules26123642)
Supplement: Supplementary file 1 [file molecules-26-03642-s001.zip › molecules-1204291-supplementary.pdf]

Supplementary Materials

Ca^+ ions solvated in helium clusters

Massimiliano Bartolomei,[†] Paul Martini,[‡] Ricardo Pérez de Tudela,[¶] Tomás González-Lezana,[*,†] Marta I. Hernández,[*,†] José Campos-Martínez,[†] Javier Hernández-Rojas,[§] José Bretón,[§] and Paul Scheier[‡]

†*Instituto de Física Fundamental (IFF-CSIC), Serrano 123, Madrid 28006, Spain*

‡*Institut für Ionenphysik und Angewandte Physik, Universität Innsbruck, Technikerstr. 25, A-6020 Innsbruck, Austria.*

¶*Lehrstuhl für Theoretische Chemie, Ruhr-Universität Bochum, 44780 Bochum, Germany*

§*Departamento de Física and IUdEA, Universidad de La Laguna, 38205 Tenerife, Spain*

E-mail: t.gonzalez.lezana@csic.es; marta@iff.csic.es

1. Methods for the calculation of cluster energies and structures

1.1 Basin-Hopping (BH)

Favorable structures for He_NCa^+ clusters, with N≤50, were found by unbiased BH global optimization technique.[1] This stochastic algorithm explores the energy landscapes[2] by performing random moves on local minima, according to Metropolis criterion.[3–9] Suitable parameters for the BH simulations such as optimization temperature and number of steps, were determined doing different preliminary tests on $He_{20}Ca^+$ cluster.

The results obtained in this work were achieved by means of four independent series of 10^4 BH steps with a fixed optimization temperature of $k_BT = 1$ meV, where k_B is the Boltzmann constant. The same global minimum was located in all trajectories.

During the BH sampling, all nonequivalent minima were stored into a database. However, the weakness of the interaction between Ca^+ ion and He atom, complicates the ordering of the classical local minima.[10–15] Two procedures were performed to achieve this ordering of the local minima. On one hand and for $N \geq 30$, we run 3 independent series of 10^4 BH steps at random temperatures between $k_BT = 2$ and 5 meV. On the other hand, the lowest 10^3 energy minimum structures found to each cluster with $N \geq 25$, were reoptimized permuting the Ca^+ ion with one Helium atom randomly.

For a given cluster size (N) and a given BH local minimum, labelled by α and with energy $U^{(N)}_{\alpha,\mathrm{BH}}$, the nuclear quantum effects were introduced in the harmonic approximation with the zero point energy (ZPE) correction, giving the (quantum) α energy as:

$$U^{(N)}_{\alpha,Q} = U^{(N)}_{\alpha,\mathrm{BH}} + 1/2 \sum_i (\hbar w^i_\alpha), \qquad (1)$$

where $w^i_\alpha(i = 1, ..., 3N - 6)$ is the i-vibrational frequency of the α minimum calculated by means of the diagonalization of the Hessian matrix.

1.2 Diffusion Monte Carlo (DMC)

Quantum ground states of the He_NCa^+ clusters were computed by means of the DMC method.[16,17] This approach involves the transformation of the time-dependent Schrödinger equation to a diffusion equation by changing the variable time, t, to imaginary time, $\tau = it$. The ground state is achieved as the remaining term in the (imaginary time) propagation of the diffusion equation. In this random-walk method, the wavefunction is represented by a set of replicas (different configurations of the particles of the system) which randomly translate

at each time step $\Delta\tau$ according to the kinetic energy term and, in addition, multiply or disappear with a probability depending on the potential energy.

We have used the code developed by Sandler and Buch[18–20] which has been successful in the study of various molecular clusters.[13,21,22] The initial population of replicas was built from a Gaussian distribution centered in the minimum geometry of the PES, previously determined from the BH calculations (for the larger clusters, other geometries were tested to look for a more rapid stabilization of the energies). In the calculations, a large time step ($\Delta\tau$= 100 a.u.) is initially used for a total propagation time T = 15 $\times 10^5$ a.u. (in order to first explore the configuration space and reach an initial stabilization of the energy), followed by three sucessive propagations (T =7.5 $\times 10^5$ a.u.) using shorter time steps ($\Delta\tau$=50, 25 and 12.5 a.u.). For each of the four propagations, the ground state energy was estimated by averaging during the last two thirds of the propagation, thus allowing us to study the time step dependence of the energies. In addition, dependence of energies with the number of replicas, N_r, was analyzed by repeating the calculations using various sizes, typically N_r= 2500, 5000, 10000, 20000, and 25000. Also, for each cluster size, about 6-8 independent runs were carried out from which an average energy and a standard deviation was obtained. As an example, dependence of the $He_{26}Ca^+$ energies with $\Delta\tau$ and N_r is depicted in Fig. S1. On the one hand, it can be seen that dependence with the time step is quite weak, the standard deviations being larger than these small variations in the energies. On the other hand, energies do vary significantly with N_r and it is found that they approximately follow the behavior $E(N_r) = E_\infty + c/N_r$ noted in previous DMC studies[23] and shown in the Fig. S1 by a blue line. These analyses have allowed us to determine optimum values of N_r depending of the cluster sizes: for $N < 11$, $11 \leq N < 17$, $17 \leq N \leq 27$, $N > 27$ we chose N_r= 10000, 20000, 25000 and 30000, respectively. In this way, energies were easily converged for the smaller clusters ($N < 17$). For larger sizes, calculations turned out rather challenging due to an increasing artificial evaporation of He atoms (separation of He atoms from the cluster for some replicas) and for this reason, report of DMC energies in the main article is restricted to the range of small clusters. Nevertheless, we were able to partially mitigate this problem by setting a radius of confinement (barrier in the He-Ca^+ interaction) at 7.5 Å, for N =17-19, steadily increasino up to 9.5 Å for N = 26-30. Cluster energies obtained in this way are reported in Table S1, together with their corresponding standard deviations.

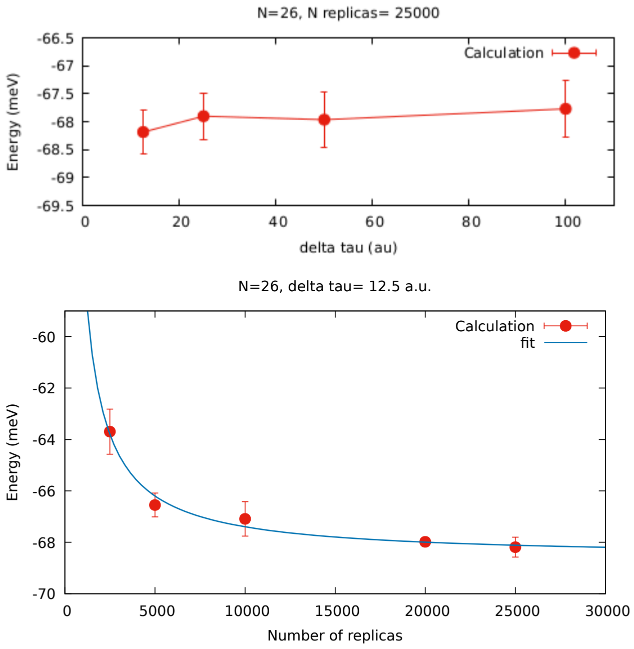

Figure S1: Upper panel: Dependence of the energy of $He_{26}Ca^+$ with the time step, using N_r=25000. Lower panel: Dependence of the cluster energy with the number of replicas N_r, for $\Delta\tau$= 12.5 a.u.; in blue line, analytical fit (see text).

Probability density distributions for the He-Ca^+ and He-He distances were obtained by means of the descendant weighting algorithm,[17] using nine generations for each DMC run. In the method, a weight proportional to the number of descendants is assigned to each replica and, in this way, a reasonable estimation of the squared wavefunction can be obtained.[19]

1.3 Path Integral Monte Carlo (PIMC)

The PIMC method is the same as in previous applications for the study of similar ion doped helium clusters[13,15] and it has been described elsewhere before.[24] In this approach the overall density matrix of the system at a given temperature T is expressed in terms of the product of densities at different values of the temperature $T' = T \times M$, which are evaluated on a set of position vectors $\mathbf{r}_i^\alpha$ of the particles forming the cluster, $\mathcal{R}_\alpha \equiv \{\mathbf{r}_1^\alpha, ..., \mathbf{r}_N^\alpha\}$. The index α runs over the so-called M quantum beads. The energy of each cluster is estimated by means of the thermodynamic approach developed by Barker:[25]

$$\langle E \rangle_{\text{thermo}} = \frac{3N}{2\tau} - \left\langle \sum_{\alpha=0}^{M-1} \sum_{i=1}^{N} \frac{(\mathbf{r}_i^\alpha - \mathbf{r}_i^{\alpha+1})^2}{4M\lambda_m \tau^2} - V \right\rangle, \qquad (2)$$

where $\lambda_m = \hbar^2/2m$, m is the mass of He or Ca^+ and $\tau = \beta/M$, with $\beta = (k_B T)^{-1}$. The expression in Eq. (2) consists on a first term describing the classical kinetic energy multiplied by M (the number of quantum beads in the factorization of the density matrix[13,15]) and a second term with the average of the energy due to the spring-like interaction assumed between consecutive beads in the same ring describing a specific particle and of the potential energy V. The PIMC calculations, performed at $T = 1$ K and with $M = 200$ quantum beads (moved in groups of 8 beads following a staging method[26,27]), have been found enough to ensure convergence (see Fig. S2). No confinement has been imposed to the movement of He atoms and the inspection of the corresponding probability density functions proved that in fact it is not necessary. Analogously, the boson exchange has not been included in the simulation. Neither the temperature considered in our calculation is low enough nor the helium coverage around the ion sizes considered in our theoretical study (up to $N = 50$ He atoms) is sufficient to expect dramatic effects due to the inclusion of this symmetry. In fact, the PIMC study by Galli *et al.*[28] on He clusters doped with alkali and alkali-earth ions, He_N-X^+ performed also at $T = 1$ K revealed that radial ^{4}He densities around a Na^+ and Mg^+ ions with distinguishable and indistinguishable He atoms were almost identical for $N = 64$ (see Fig. 3 of such a reference) and differences in the energies were not larger than 0.007 meV in any case. Given that the Mg^+-He interaction potential is similar to that of Ca^+-He considered here, we can presume that neglecting the boson exchange does not affect substantially to the final results.

Cluster energies computed in this way are reported in Table S1, compared with the DMC ones.

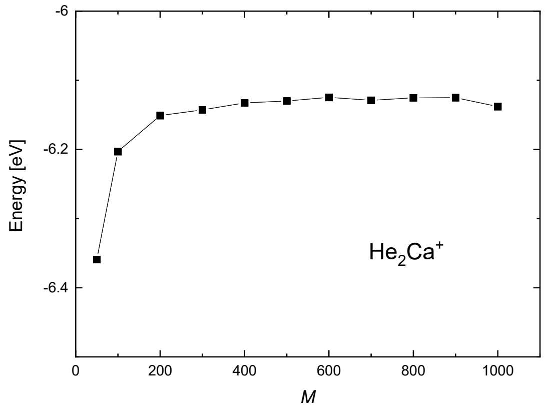

Figure S2: Dependence of the PIMC energy of He_2Ca^+ with the number of quantum beads, M. The value of the energy obtained at $M = 200$ differs less than 0.5 % with respect to the result for larger Ms.

1.4 PIMC and DMC energies

TABLE S1: He_NCa^+ energies E_N and standard deviations (in parenthesis), in meV, as obtained from the PIMC and DMC approaches.

N	PIMC	DMC	N	PIMC	DMC
1	-3.00 (0.01)	-3.13 (0.01)	26	-71.54 (0.10)	-68.19 (0.39)
2	-6.15 (0.01)	-6.30 (0.01)	27	-72.61 (0.11)	-69.24 (0.78)
3	-9.33 (0.01)	-9.49 (0.01)	28	-73.65 (0.09)	-70.15 (0.69)
4	-12.53 (0.02)	-12.69 (0.03)	29	-74.68 (0.10)	-70.98 (0.53)
5	-15.73 (0.03)	-15.94 (0.02)	30	-75.71 (0.09)	-72.01 (0.45)
6	-18.93 (0.03)	-19.16 (0.02)	31	-76.72 (0.10)	—-
7	-22.15 (0.03)	-22.42 (0.04)	32	-77.77 (0.11)	—-
8	-25.36 (0.04)	-25.72 (0.03)	33	-78.76 (0.12)	—-
9	-28.60 (0.03)	-28.97 (0.04)	34	-79.82 (0.11)	—-
10	-31.82 (0.03)	-32.22 (0.02)	35	-80.84 (0.10)	—-
11	-35.10 (0.03)	-35.48 (0.05)	36	-81.88 (0.11)	—-
12	-38.36 (0.02)	-38.68 (0.04)	37	-82.92 (0.12)	—-
13	-41.58 (0.02)	-41.86 (0.09)	38	-83.98 (0.13)	—-
14	-44.77 (0.02)	-44.92 (0.09)	39	-85.03 (0.12)	—-
15	-47.88 (0.03)	-47.90 (0.18)	40	-86.07 (0.13)	—-
16	-50.92 (0.02)	-50.77 (0.30)	41	-87.06 (0.12)	—-
17	-53.86 (0.03)	-53.55 (0.17)	42	-88.21 (0.15)	—-
18	-56.61 (0.03)	-55.86 (0.13)	43	-89.23 (0.14)	—-
19	-59.19 (0.03)	-58.07 (0.26)	44	-90.24 (0.14)	—-
20	-61.72 (0.04)	-59.95 (0.35)	45	-91.39 (0.16)	—-
21	-63.90 (0.04)	-61.65 (0.52)	46	-92.39 (0.16)	—-
22	-65.86 (0.06)	-63.38 (0.37)	47	-93.45 (0.17)	—-
23	-67.50 (0.06)	-64.63 (0.28)	48	-94.48 (0.17)	—-
24	-69.18 (0.09)	-66.05 (0.57)	49	-95.54 (0.15)	—-
25	-70.41 (0.07)	-67.28 (0.33)	50	-96.64 (0.18)	—-

2. Additional Figures

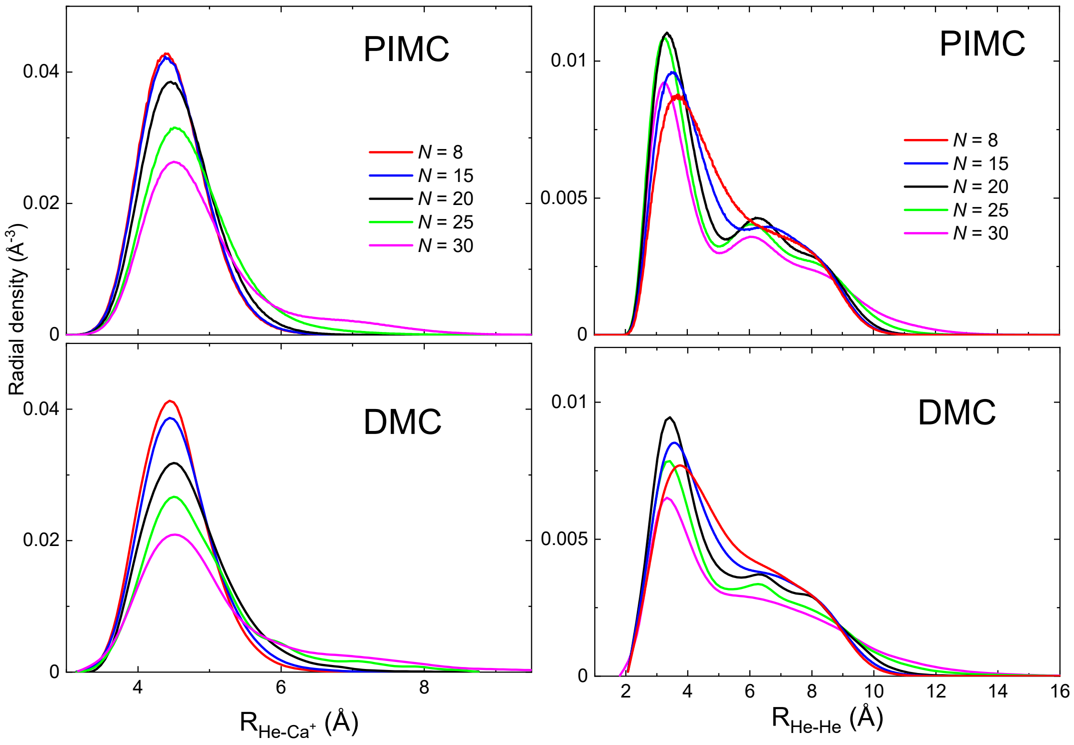

Figure S3: **Cluster radial densities (Å^{-3}) as functions of the He-Ca$^+$ (left panels) and He-He (right panels) distances (Å) obtained by means of the PIMC (top panels) and DMC (bottom panels) approaches, for different sizes of He$_N$Ca$^+$: N = 8 (red), 15 (blue), 20 (black), 25 (green) and N = 30 (pink).** For the radial densities vs. the He-Ca$^+$ distance, it can be seen that DMC distributions generally agree with the more accurate PIMC ones (already presented in the main article, Fig.6). Regarding the dependence of the radial densities with the He-He distance, PIMC distributions show a maximum near $R_{\text{He-He}} \sim 3.5$ Å and a broad shoulder between 5 and 10 Å which is more structured for N= 20, 25 and 30. In addition, for $N = 25$ and 30, the distribution becomes more extended in the region of larger He-He distances, in consistency with the formation of a second shell. DMC densities (right-lower panel) qualitatively agree with the PIMC ones.

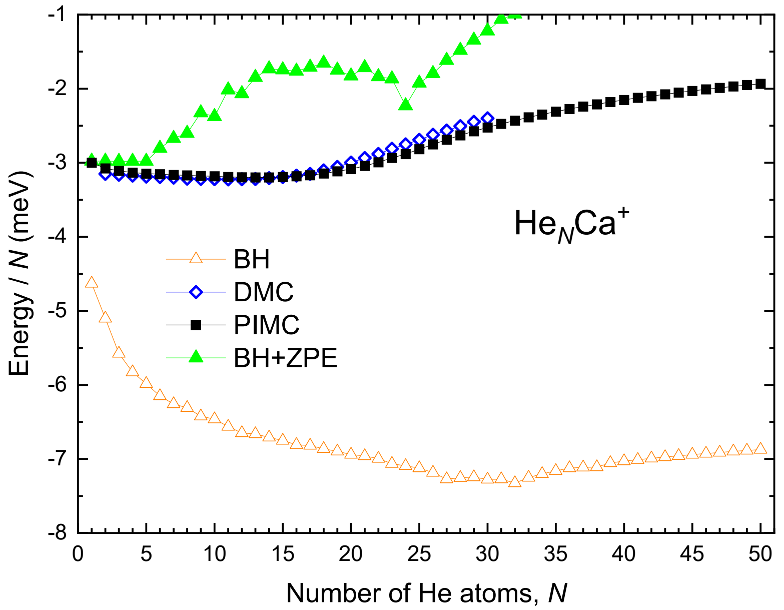

Figure S4: **Energy per He atom obtained by means of the BH (open orange triangles), BH+ZPE (green triangles), DMC (open blue diamonds) and PIMC (black squares) approaches.** DMC energies per atom compare well with PIMC ones but tend to increase more rapidly for $N > 16$, probably due to an increasing artificial evaporation (see Sec. I.B of this document). BH energies are much lower that the energies obtained via the DMC or PIMC approaches, thus indicating the important role in these systems of the nuclear quantum effects. The BH+ZPE estimation only compares well with the quantum energies for the lightest clusters, $N \leq 5$. The extremely weak and anharmonic He-Ca$^+$ and He-He potentials and the presence of a large number of shallow local potential energy minima seems to be the reason which jeopardizes a good performance of the ZPE correction.

References

References

(1) Wales, D. J.; Doye, J. P. K. Global optimization by basin-hopping and the lowest energy structures of Lennard-Jones clusters containing up to 110 atoms. *J. Phys. Chem. A* **1997**, *101*, 5111–5116.

(2) Wales, D. J. *Energy Landscapes*; Cambridge University Press: Cambridge, 2003.

(3) Hernández-Rojas, J.; Wales, D. J. Global minima for rare gas clusters containing one alkali metal ion. *J. Chem. Phys.* **2003**, *119*, 7800–7804.

(4) Hernández-Rojas, J.; Bretón, J.; Gomez Llorente, J. M.; Wales, D. J. Lowest-energy structures of $(C_{60})_nX$ ($X=Li^+,Na^+,K^+,Cl^-$) and $(C_{60})_nYCl$ (Y=Li,Na,K) clusters for $n \leq 13$. *J. Chem. Phys.* **2004**, *121*, 12315–12322.

(5) Hernández-Rojas, J.; Bretón, J.; Gomez Llorente, J. M.; Wales, D. J. Global minima of $(C_{60})_nCa^{2+}$, $(C_{60})_nF^-$ and $(C_{60})_nI^-$ clusters. *Chem. Phys. Lett.* **2005**, *410*, 404 – 409.

(6) Hernández-Rojas, J.; Bretón, J.; Gomez Llorente, J. M.; Wales, D. J. Global Potential Energy Minima of $C_{60}(H_2O)_n$ Clusters. *J. Phys. Chem. B* **2006**, *110*, 13357–13362.

(7) Hernández-Rojas, J.; Calvo, F.; Rabilloud, F.; Bretón, J.; Gomez Llorente, J. M. Modeling Water Clusters on Cationic Carbonaceous Seeds. *J. Phys. Chem. A* **2010**, *114*, 7267–7274.

(8) Hernández-Rojas, J.; Calvo, F.; Bretón, J.; Gomez Llorente, J. M. Confinement Effects on Water Clusters Inside Carbon Nanotubes. *J. Phys. Chem. C* **2012**, *116*, 17019–17028.

(9) Acosta-Gutiérrez, S.; Bretón, J.; Hernández-Rojas, J.; Gomez Llorente, J. M. Optimal covering of C_{60} fullerene by rare gases. *J. Chem. Phys.* **2012**, *137*, 074306.

(10) Calvo, F.; Naumkin, F.; Wales, D. Nuclear quantum effects on the stability of cationic neon clusters. *Chem. Phys. Lett.* **2012**, *551*, 38 – 41.

(11) Rodríguez-Cantano, R.; Pérez de Tudela, R.; Bartolomei, M.; Hernández, M. I.; Campos-Martínez, J.; González-Lezana, T.; Villarreal, P.; Hernández-Rojas, J.; Bretón, J. Coronene molecules in helium clusters: Quantum and classical studies of energies and configurations. *J. Chem. Phys.* **2015**, *143*, 224306.

(12) Bartolomei, M.; Pérez de Tudela, R.; Arteaga, K.; González-Lezana, T.; Hernández, M. I.; Campos-Martínez, J.; Villarreal, P.; Hernández-Rojas, J.; Bretón, J.; Pirani, F. Adsorption of molecular hydrogen on coronene with a new potential energy surface. *Phys. Chem. Chem. Phys.* **2017**, *19*, 26358–26368.

(13) Rastogi, M.; Leidlmair, C.; An der Lan, L.; Ortiz de Zárate, J.; Pérez de Tudela, R.; Bartolomei, M.; Hernández, M. I.; Campos-Martínez, J.; González-Lezana, T.; Hernández-Rojas, J. et al. Lithium ions solvated in helium. *Phys. Chem. Chem. Phys.* **2018**, *20*, 25569–25576.

(14) Calvo, F.; Hamdi, R.; Mejrissi, L.; Oujia, B. Questioning the structure of Sr+Arn clusters. *Eur. Phys. J. D.* **2018**, *72*, 133.

(15) Pérez de Tudela, R.; Martini, P.; Goulart, M.; Scheier, P.; Pirani, F.; Hernández-Rojas, J.; Bretón, J.; Ortiz de Zárate, J.; Bartolomei, M.; González-Lezana, T. et al. A combined experimental and theoretical investigation of Cs^+ ions solvated in He_N clusters. *J. Chem. Phys.* **2019**, *150*, 154304.

(16) Anderson, J. B. A Random–Walk Simulation of the Schrödinger Equation: H_3^+. *J. Chem. Phys.* **1975**, *63*, 1499–1503.

(17) Suhm, M. A.; Watts, R. O. Quantum Monte Carlo Studies of Vibrational States in Molecules and Clusters. *Phys. Rep.* **1991**, *204*, 293 – 329.

(18) Buch, V. Treatment of Rigid Bodies by Diffusion Monte-Carlo. Application to the Para-H_2...H_2O and Ortho-H_2...H_2O Clusters. *J. Chem. Phys.* **1992**, *97*, 726–729.

(19) Sandler, P.; Buch, V.; Sadlej, J. Ground and excited states of the complex of CO with water: A diffusion Monte Carlo study. *J. Chem. Phys.* **1996**, *105*, 10387–10397.

(20) Sandler, P.; Buch, V. *General purpose QCLUSTER program for Rigid Body Diffusion Monte Carlo simulation of an arbitrary molecular cluster*; private communication, 1999.

(21) Kolmann, S. J.; D'Arcy, J. H.; Jordan, M. J. T. Quantum Effects and Anharmonicity in the H_2-Li^+-benzene complex: A Model for Hydrogen Storage Materials. *J. Chem. Phys.* **2013**, *139*, 234305.

(22) Ortiz de Zárate, J.; Bartolomei, M.; González-Lezana, T.; Campos-Martínez, J.; Hernández, M. I.; Pérez de Tudela, R.; Hernández-Rojas, J.; Bretón, J.; Pirani, F.; Kranabetter, L. et al. Snowball formation for Cs^+ solvation in molecular hydrogen and deuterium. *Phys. Chem. Chem. Phys.* **2019**, *21*, 15662–15668.

(23) Slavíček, P.; Lewerenz, M. Snowballs, quantum solvation and coordination: lead ions inside small helium droplets. *Phys. Chem. Chem. Phys.* **2010**, *12*, 1152–1161.

(24) Rodríguez-Cantano, R.; González-Lezana, T.; Villarreal, P. Path integral Monte Carlo investigations on doped helium clusters. *Int. Rev. Phys. Chem.* **2016**, *35*, 37–68.

(25) Barker, J. A. A quantum-statistical Monte Carlo method; path integrals with boundary conditions. *J. Chem. Phys.* **1979**, *70*, 2914.

(26) Ceperley, D. M. Path integrals in the theory of condensed helium. *Rev. Mod. Phys.* **1995**, *67*, 279.

(27) Pollock, E. L.; Ceperley, D. M. Simulation of quantum many-body systems by path-integral methods. *Phys. Rev. B* **1984**, *30*, 2555–2568.

(28) Galli, D. E.; Ceperley, D. M.; Reatto, L. Path integral Monte Carlo study of ^{4}He clusters doped with alkali and alkali-earth ions. *J. Phys. Chem. A* **2011**, *115*, 7300–7309.
